# COVID-19 Infection Presenting With Cheilitis and Fever in a Toddler

**DOI:** 10.7759/cureus.12444

**Published:** 2021-01-03

**Authors:** La Nyka A Christian, Jaya Wadhawan, Sally Abdelmalek, Louisdon Pierre, Adebayo Adeyinka

**Affiliations:** 1 Pediatrics, The Brooklyn Hospital Center, Brooklyn, USA

**Keywords:** sars cov-2, cheilitis, covid 19, high fever, pediatric multi-system inflammatory syndrome, multi-system inflammatory disease in children (mis-c)

## Abstract

Coronavirus disease 2019 (COVID-19) is a newly found infectious disease caused by severe acute respiratory syndrome coronavirus 2 (SARS-CoV-2), first observed in Wuhan, China, in December 2019. An otherwise healthy 13-month-old male presented with persistent fever and cheilitis as his initial findings of COVID-19 in April 2020 prior to the discovery and classification of the multisystem inflammatory syndrome in children (MIS-C). Clinical symptoms of COVID-19 are still evolving in the pediatric population, ranging from being asymptomatic to varied symptoms, such as fever, abdominal pain, and myocarditis. Other manifestations such as conjunctivitis and cheilitis can offer clues. We speculate that cheilitis can be a sign of the hyperinflammatory state, as seen in MIS-C.

## Introduction

According to the American College of Emergency Physicians, fever is the most commonly encountered chief complaint to the emergency department in the United States, accounting for 15% of visits [1]. The recent outbreak of coronavirus disease 2019 (COVID-19) in the US and across the world has resulted in global catastrophe with innumerable deaths and hospitalizations. COVID-19 is asymptomatic in a number of children, and its varied manifestations are still being elucidated. Cheilitis has not been previously described as a clinical presentation of COVID-19. Cheilitis refers to the inflammation of the lips causing a dry, cracked appearance. It is found on the peri-oral region, vermilion border, or labial mucosa. There are many different types of this condition, such as cheilitis simplex, angular, contact, exfoliative, drug-induced, actinic, glandular, and plasma cell [2]. The most common type is cheilitis simplex that is characterized by the dry, cracked appearance with peeling of the lips, which occurs due to a lack of moisture due to excessive lip licking or smacking [3]. We describe a patient with fever and cheilitis diagnosed with clinical pneumonia in the setting of COVID-19 infection.

## Case presentation

A 13-month-old male presented to our pediatric emergency department (ED) in early April 2020 with the chief complaint of fussiness, decreased appetite, and fever for five days. His mother reported that there was no resolution of his fever despite the use of antipyretics. At the presentation time, his mother denied cough, runny nose, ear pulling, shortness of breath, rash, vomiting, or diarrhea. She reported no recent travel nor sick contacts at home. Social history was remarkable for crowded and close living conditions, with five other family members sharing a two-bedroom apartment. The patient remained isolated at home since the pandemic began, but other family members had been out of the house for groceries. 

He was nontoxic on physical exam, appearing but fussy and febrile with a temperature of 102.7°F (Table 1). Physical exam was remarkable for dry, cracked lips with fissuring along the vermilion border and labial mucosa. His mucous membranes were dry, and there was pharyngeal erythema without exudates or tonsillar hypertrophy. The chest was clear to auscultation bilaterally. The patient had no conjunctivitis, lymphadenopathy, hepatosplenomegaly, nor edema or rash on the trunk, hands, and feet. 

**Table 1:** Vital signs of the patient on admission

Vital signs	Patient	Normal range for age
Temperature	102.7° F	95-100.3° F
Heart rate	172 beats per minute	80-130 beats per minute
Respiratory rate	32 breaths/min	40-60 breaths/min
Oxygen saturation on room air	98%	94-100%

Laboratory findings showed a negative respiratory viral panel, a normal complete blood count (CBC) with lymphocytic predominance (Table 2), elevated C-reactive protein (CRP) of 25.81 mg/dL. The comprehensive metabolic panel (CMP) revealed metabolic acidosis with a normal anion gap with ketones present in the urinalysis. We obtained blood and urine cultures, and the patient was admitted and started empirically on ceftriaxone, antipyretics, and intravenous fluids. 

**Table 2:** Complete blood count of the patient WBC - white blood cells; RBC - red blood cells; Hgb - hemoglobin; Hct - hematocrit; MCV - mean corpuscular volume; MCH - mean corpuscular hemoglobin; MCHC - mean corpuscular hemoglobin concentration; RDW - red cell distribution width

Complete blood count	Result	Reference range (for patient age)	Unit
WBC	7.7	6.0-17.5	x 10^9^/L
RBC	4.46	3.8-5.4	x 10¹²/L
Hgb	11.4	13.1-15.5	g/L
Hct	35	39-47	L/L
MCV	77	72.0-88.0	fL
MCH	26	26-34	ρg
MCHC	329	320-360	g/L
RDW	12.9	11.5-16.0	%
Platelet	357	130-400	x 10^9^/L
Neutrophil	2.5	1.5-8.5	x 10^9^/L
Lymphocytes	3.9	3-13.0	x 10^9^/L
Monocytes	1.1	0.1-1.9	x 10^9^/L
Basophils	0.2	0.0-0.3	x 10^9^/L
Eosinophils	0	0.0-1.5	x 10^9^/L

**Figure 1:**
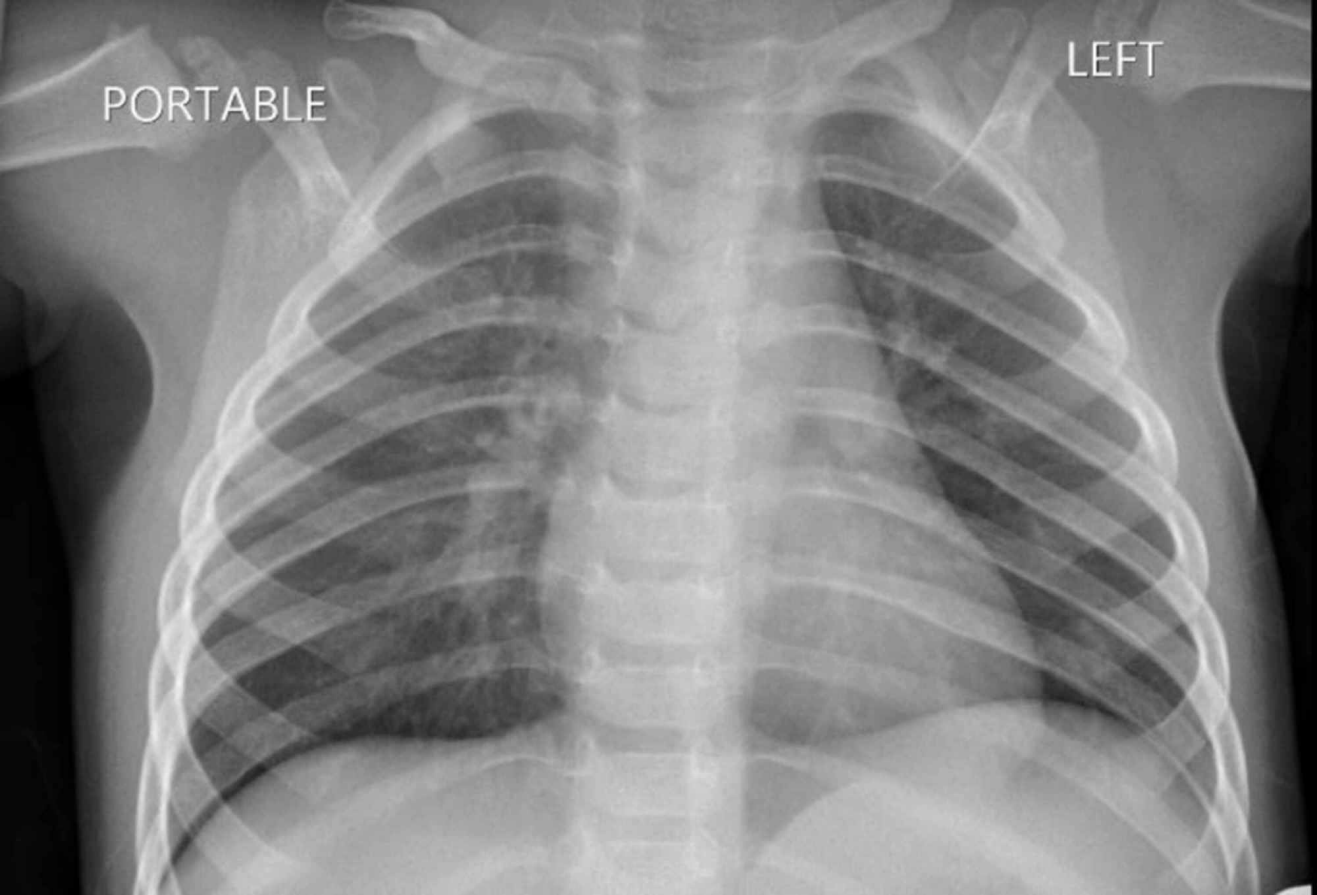
Portable CXR: bilateral interstitial infiltrates more prominent on the right perihilar area, which could represent atypical pneumonia CXR - chest X-ray

Due to persistent fever, a chest X-ray (CXR) was ordered, which showed bilateral interstitial infiltrates more prominent on the right peri-hilar area, which could represent atypical pneumonia (Figure 1). Urine and blood cultures were negative. However, the severe acute respiratory syndrome coronavirus 2 (SARS-COV-2) polymerase chain reaction (PCR) was positive 24 hours after admission to the hospital. The patient was subsequently started on azithromycin for clinical suspicion of atypical pneumonia related to COVID-19, correlating with the CXR findings. The fever subsided after the first day of antibiotics, and the cheilitis improved during the admission. The patient was discharged home with isolation guidelines and completed five days of azithromycin and seven days of amoxicillin clavulanate for suspected clinical pneumonia. 

## Discussion

COVID-19 is a novel viral illness caused via close contact, respiratory droplets, and aerosolized particles [4, 5]. In the review of the current literature, children appear to display milder clinical manifestations for COVID-19 compared to the adult population [6]. More recently, SARS-CoV-2 virus infections have been associated with Kawasaki like illness with a hyperimmune state and described as a multisystem inflammatory syndrome of children (MIS-C) [7]. 

The Centers for Disease Control and Prevention (CDC) noted that 56% of laboratory-confirmed COVID-19 pediatric cases presented with fever, 54% with cough, and 13% with shortness of breath. Other associated findings are gastrointestinal symptoms, including abdominal discomfort, nausea, vomiting, and diarrhea [8]. Laboratory COVID-19 confirmed pediatric cases can show normal total white blood cell counts with lymphopenia, elevated liver enzymes, lactate dehydrogenase, C-reactive protein, erythrocyte sedimentation rates, and pro-calcitonin levels [9, 10]. 

Pediatric cases of persistent fever and cheilitis often coincide in children with Kawasaki disease. Other associated signs and symptoms are bilateral non-exudative conjunctivitis, cervical lymphadenopathy, mucositis, polymorphous rash, and edema of the hands and feet, all absent in our patient. At the time of our literature review, there have been 15 reported cases of atypical, typical, and incomplete Kawasaki like disease in children in the New York State as of April 29th, 2020. Over 50% of these patients with MIS-C presented in shock and required blood pressure support, while 33% required mechanical ventilation. As of the date of this article, no fatalities were reported [7]. The exact mechanism of COVID-19 infection and this new multisystem inflammatory syndrome remains unclear. 

As of April 2020, the World Health Organization (WHO) noted that 80% of confirmed COVID-19 positive pediatric patients displayed mild symptoms such as fever, non-productive cough, and fatigue and often recovered without requiring hospitalization [11]. This patient presented with fever without an apparent source prompting a partial workup for sepsis. The patient required hospitalization for continued diagnostic workup and empiric treatment. This patient did not develop any respiratory complications from his COVID-19 infection. It is important to now consider COVID-19 as a potential differential diagnosis in addition to routine sepsis workup in pediatric patients presenting with persistent fever without a defined source of infection. Occult infectious etiologies such as bacteremia, urinary tract infection, and meningitis, should not be discounted for patients with persistent fever. Acute cheilitis may be another manifestation of COVID-19, and the occurrence of this finding in febrile children should raise the suspicion of COVID- 19. We suggest testing for COVID-19 for febrile children presenting with cheilitis that is otherwise unexplained. 

## Conclusions

New evidence suggests that more severe presentations associated with COVID-19 are emerging in pediatric patients, MIS-C, which can present with a prolonged history of fever, along with other criteria. In cases where children present with prolonged fever of unknown origin, they should continue to receive a workup for sepsis in addition to considering COVID-19 infection during this pandemic. MIS-C should be also be considered as a possibility. Cheilitis may be a new sign or clinical manifestation of COVID-19. Although the occurrence of cheilitis has not been well described in the current COVID-19 literature, more research is needed to investigate this finding. 
